# IoT Sensor Networks in Smart Buildings: A Performance Assessment Using Queuing Models

**DOI:** 10.3390/s21165660

**Published:** 2021-08-23

**Authors:** Brena Santos, André Soares, Tuan-Anh Nguyen, Dug-Ki Min, Jae-Woo Lee, Francisco-Airton Silva

**Affiliations:** 1 Programa de Pós-Graduação em Ciência da Computação, Universidade Federal do Piauí (UFPI), Teresina-Piauí 64049-550, Brazil; brenamaia@ufpi.edu.br (B.S.); andre.soares@ufpi.edu.br (A.S.); faps@ufpi.edu.br (F.-A.S.); 2 Konkuk Aerospace Design-Airworthiness Research Institute (KADA), Konkuk University, Seoul 05029, Korea; anhnt2407@konkuk.ac.kr; 3 Department of Computer Science and Engineering, College of Engineering, Konkuk University, Seoul 05029, Korea; dkmin@konkuk.ac.kr; 4 Department of Aerospace Information Engineering, Konkuk University, Seoul 05029, Korea

**Keywords:** internet of things (IoT), smart building, queuing model, sensors networks

## Abstract

Smart buildings in big cities are now equipped with an internet of things (IoT) infrastructure to constantly monitor different aspects of people’s daily lives via IoT devices and sensor networks. The malfunction and low quality of service (QoS) of such devices and networks can severely cause property damage and perhaps loss of life. Therefore, it is important to quantify different metrics related to the operational performance of the systems that make up such computational architecture even in advance of the building construction. Previous studies used analytical models considering different aspects to assess the performance of building monitoring systems. However, some critical points are still missing in the literature, such as (i) analyzing the capacity of computational resources adequate to the data demand, (ii) representing the number of cores per machine, and (iii) the clustering of sensors by location. This work proposes a queuing network based message exchange architecture to evaluate the performance of an intelligent building infrastructure associated with multiple processing layers: edge and fog. We consider an architecture of a building that has several floors and several rooms in each of them, where all rooms are equipped with sensors and an edge device. A comprehensive sensitivity analysis of the model was performed using the Design of Experiments (DoE) method to identify bottlenecks in the proposal. A series of case studies were conducted based on the DoE results. The DoE results allowed us to conclude, for example, that the number of cores can have more impact on the response time than the number of nodes. Simulations of scenarios defined through DoE allow observing the behavior of the following metrics: average response time, resource utilization rate, flow rate, discard rate, and the number of messages in the system. Three scenarios were explored: (i) scenario A (varying the number of cores), (ii) scenario B (varying the number of fog nodes), and (iii) scenario C (varying the nodes and cores simultaneously). Depending on the number of resources (nodes or cores), the system can become so overloaded that no new requests are supported. The queuing network based message exchange architecture and the analyses carried out can help system designers optimize their computational architectures before building construction.

## 1. Introduction

The Internet of Things (IoT) has emerged as a dominant computing paradigm to enable ubiquitous connectivity between different “things” [1]. The IoT connects any item to the Internet through distributed sensors for identification, positioning, tracking, monitoring, and management [2,3]. The number of IoT devices worldwide is predicted to nearly double from 8.74 billion in 2020 to over 16.44 billion in 2025 [4]. IoT holds promise because of its importance in many commerce, industry, and education applications [5]. IoT devices can be used, for example, to automate activities in smart homes [6], traffic from vehicular networks [7], and health monitoring of elderly in their homes [8]. Therefore, an IoT system grants services to smart scenarios in different contexts, efficiently managing hardware, software, and communication resources to reduce costs in specific domains. Identifying abnormal behavior in a monitored environment using IoT has been very useful in smart homes and buildings.

According to the United Nations, the world’s urban population is projected to grow by 2.5 billion from 2014 to 2050, when it will account for 66% of the total global population [9]. The growing population in cities increases the demand for the fundamental needs of the people living there, such as housing, utilities, medical care, welfare, education, and employment [10]. The smart city concept has been envisioned to deal with challenges faced during the growth of cities. A smart city denotes “the effective integration of physical, digital and human systems in the built environment to deliver a sustainable, prosperous and inclusive future for its citizens” [11]. As the cells of smart cities, smart buildings integrate intelligence, enterprise, control, materials, and construction to advance the building’s energy efficiency, longevity, comfort, and satisfaction [12]. In both the smart cities and buildings contexts, the “smart” refers to the development, integration, and utilization of intelligent systems based on Information and Communication Technologies (ICT). Originally, smart home technology was used to control environmental systems such as lighting and heating. However, for some time now, IoT has allowed almost all house electrical components to be connected to a central system, making it possible to monitor the environment. A smart home can provide services based on user needs. From the concept of smart homes, IoT can be extended to a broader context: smart buildings. Managing smart buildings requires more sophisticated computing infrastructures and a readiness to meet a great demand for data generation. The computational power of cloud computing is used to process such demand for a long time.

Cloud computing has been the backbone for hosting and offering subscription-oriented computing and application services. It is also used to execute the applications for different IoT-enabled cyber-physical systems [13]. Using cloud computing is sometimes impossible by the distant placement of IoT devices. Edge and fog computing emerged to place services closer to the data source [14]. The central research problem in this work is that such addition of processing layers in the IoT systems architecture requires performance evaluations from the earliest stages of development. However, evaluations with real experiments can be costly because there are many configuration possibilities. Simple home automation with IoT devices can cost more than a hundred thousand dollars (Homeadvisor Service: https://www.homeadvisor.com/cost/electrical/install-or-repair-a-home-automation-system/, accessed on 2 July 2021) Unnecessary expenses can be made to obtain resources that will not be used. Analytical models can be a solution in this context, allowing predictions based on probabilistic calculations [15,16,17,18,19,20,21,22,23]. Queuing theory is a popular mathematical method for analyzing different systems and observing their behavior concerning system performance. Queue models are simple, didactic and efficient [24,25,26]. Queue theory applications generally have two goals: predicting system performance and finding a system design to improve performance in the planning phase [27].

Some previous work in the literature developed analytical models to assess the capabilities of IoT systems in smart homes and buildings. Some studies focused on ensuring only the functioning of the system [28,29,30,31], others analyzed the energy efficiency [30,32,33]. In particular, Zheng et al. [34] model the smart home workflow system based on the Zig-Bee and discusses the resource flow situation in the smart home system for analyzing the user comfort perception. Lin et al. [35] propose a localization approach that utilizes the neighbor received signal strength to build a fingerprint database and adopts a Markov-chain prediction model to assist positioning. Casado et al. [36] develop a control model that is applied to ensure reliable temperature monitoring and control in a smart supermarket. The efficiency of the presented approach is verified with the results obtained in the case study carried out. Ajao et al. [6] analyzed the system performance in terms only of response time and drop rate. They explored the very specific context of a window automated control, which is not a critical situation. In our case, we envision aspects related to sensors with critical latency requirements, such as fire detection, but our model can be used to represent any type of sensor in a building.

Furthermore, the previous works did not show detailed performance analyses of data transactions in the system. Very few works in literature considered these issues in a comprehensive manner, especially using queuing network models. Bouloukakis et al. in a recent work, ref. [37] presented several queuing models to represent QoS settings of IoT interactions. Nevertheless, the models are for different purposes of performance analysis without a proper consideration of system/network architecture. While, we propose in our study the adoption of a queuing network based message exchange architecture to represent the data transaction in an edge/fog infrastructure for smart buildings. Volochiy et al. in the most related work, ref. [38] proposed a queuing network for availability and safety assessment of data services in a general IoT infrastructure. We extensively propose a comprehensive queuing network based message exchange architecture to capture the data transaction in a specific IoT sensor network for smart buildings for the sake of performance evaluation. Our study presents a significant progress and contribution compared to the work [38], as well as many other above-mentioned works in the performance assessment of IoT sensor networks in smart buildings using queuing networks.

Among the above-cited papers, none explored layers of edge and fog. Previous work has also not explored the analysis of the impact of resource capacity variation on system performance. Furthermore, this work considered sensors grouped by location, an essential characteristic when monitoring more than one environment. Therefore, this paper proposes a queuing network based message exchange architecture to evaluate IoT systems for smart buildings supported by fog-edge. The contributions of this paper are as follows:

- A queuing network-based message exchange architecture, which is a useful tool for system designers to evaluate the performance of architectures for smart buildings supported by fog edge, even before their implementation. The model allows configuring parameters according to the designer’s need, including the number of nodes, service times, queue size, among others. The designer will be able to analyze various performance metrics, for example, the mean response time (MRT) and drop rate.
- A comprehensive sensitivity analysis with Design of Experiments (DoE), which allows you to analyze different factors and how changes in their levels impact the performance of a smart building system. According to Raj Jain [39], parameters on an experiment or simulation are a variable that their configured values impact the system somehow but are adjusted to be constant (e.g., Operating System). Factors are varied during the test to observe their specific impact on the system (e.g., number of cores in a server). Four factors are observed in this work, are (i) service time, (ii) number of fog nodes, (iii) number of processing cores, and (iv) queue size.
- Three simulations were carried out considering different scenarios, which serve to analyze the performance of an intelligent building: scenario A (varying the number of cores), scenario B (varying the number of fog nodes), and scenario C (varying the nodes and cores simultaneously). The monitored scenarios analyze the system’s behavior by changing the number of fog nodes and cores. Metrics such as MRT, resource utilization, drop rate, and flow rate were considered. According to the sensitivity analysis, the simulations were carried out considering the factors with the greatest impact on performance: number of cores and number of nodes. In scenarios A and B, the arrival rate was varied from 0.04 to 0.08 msg/ms. Depending on the number of resources (nodes or cores), the system can become so overloaded that no new requests are supported. For example, in scenario A, with 4 and 8 cores, the resource utilization does not exceed 80. In scenario B, when there are only two nodes in the fog, there are always dropped requests (ranging from 0.1 msg/ms to 0.35 msg/ms dropped). Scenario C indicated that by varying the parameters similarly (cores and nodes), the cores have a much greater impact on performance than the number of nodes.

The remainder of this paper is organized as follows: Section 2 shows a brief background of queuing theory. Section 3 presents the related works, comparing them with our proposal. Section 4 presents the methodology applied to carry out this work. Section 5 describes the architecture that was used as the basis for building the model. In Section 6 the proposed queuing network based message exchange architecture is presented. Section 7 describes experiments performed with Design of Experiments (DoE). Section 8 presents the results obtained from the simulations carried out. Finally, Section 9 traces some conclusions and future work.

## 2. Queuing Theory Background

**Figure 1:**
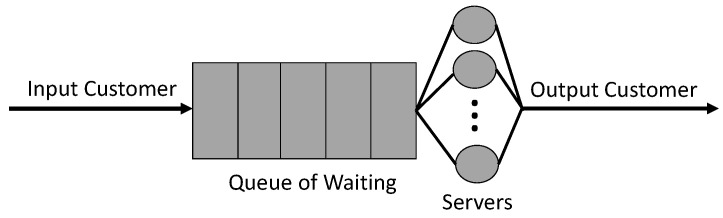
Service center (queue of waiting + one or many servers).

A queue is the implementation of a waiting list of jobs in order to obtain a service. One or more servers may provide services. This set (queue + one or more servers capable of providing services) is called the service center. If an analogy with the real world is performed, one can imagine one or more front desks accessed through a queue. All the front desks work in parallel and provide the same service, regardless of their position. In queuing theory, a service center is schematically represented as in Figure 1 [40].

A model that uses one or more service centers is called a queuing model. Several performance metrics are used to measure the performance of such a model Below, the most critical performance metrics are presented and they can characterize such a queuing model. The number of customers at a time is denoted by $n_{s}$. A random variable *n* whose mean values cannot be calculated using a probability distribution is used. The expected value for the number of customers that are at a certain time in the system is called the *n*th moment of the origin of *n* and is calculated as follows:

(1) $$E\left[n^{k}\right]=\sum_{i=0}^{\infty }i^{k}Prob\left\{n=i\right\}$$

If it is considered that *Prob*{*n* = *i*} and *E*[$n^{k}$] to be equal with averages over an infinitely long interval of time (long-run time averages), one has:

(2) $$\lim_{s\to \infty }\left(first\;s\;time\;when\;i\;customers\;are\;in\;the\;system\right)$$

(3) $$E\left[n^{k}\right]=\lim_{s\to \infty }\frac{1}{S}\int_{0}^{S}{\left[n_{u}\right]}^{k}du$$

Each client *j* that arrives in the system should spend some a time before it is served. This time is defined as the response time for customer *j* and it was noted with $r_{j}$. If it is considered that *Prob*{*r* ≤ *t*} and *E*[$r^{k}$] are equal to averages over an infinite number of customers (long-run customer averages), the following equation for the probability distribution function and expected value is obtained.

(4) $$Prob\left\{r\leq t\right\}=\lim_{j\to \infty }\left(Z\right)$$

The variable *Z* means the fraction of the first *J* customers to arrive whose response time is less or equal to *t*.

(5) $$E\left[r^{k}\right]=\lim_{j\to \infty }\frac{1}{J}\sum_{j=1}^{J}r_{j}^{k}$$

If the system is a stable one (*E*[*n*] and *E*[*r*] are finite numbers), the throughput *T* must be equal with the long-run rate for the customer arrivals, where *T* is defined as:

(6) $$T=\lim_{s\to \infty }\frac{1}{S}\left(number\;of\;clients\;departed\;in\;first\;s\;time\;units\right)$$

The time when a server is busy is called the utilization of the service center. If it is denoted by *bs*, the number of servers occupied at time *s*, U(utilization) is defined as follows:

(7) $$T=\lim_{s\to \infty }\frac{1}{S}\int_{0}^{s}b_{u}du$$

## 3. Related Work

This section presents related works with approaches similar to our proposal. Some of previous works which are related to analytical models, smart homes/buildings are considered for discussion in this section to highlight the contributions of our study.

Arbib et al. [26] propose a technique that employs the Petri net tools to model, simulate, analyze, and control at the discrete-event level the smart home applications. However, Arbib et al. did not focus on critical types of sensors but only light control, for example. Novak et al. [29] present a method for anomaly detection in user’s activities utilizing data from unobtrusive sensors. A service for a smart-home environment using this method adapts to a user’s behavior and may provide alarms to a responsible person if unusual activity is detected. As an unusual activity, they consider long periods of inactivity, lacking activity, unusual presence, and changes in daily activity patterns. Anomaly detection is based on an unsupervised classification technique Self Organizing Maps and next activity prediction employing Markov model. Novak et al. observed multiple metrics in conjunction, neither fog layer. Wang et al. [33] present an energy management modeling of a multi-source power system composed of photovoltaic (PV) array, storage, and power grid connection, and considering messages from the smart grid. The designed system can supply a tertiary building at the same time as PV may produce energy. The control strategy manages the power flow through the load concerning its power demand and public grid constraints. Wang et al. only observe energy metrics.

Nabih et al. [28] specifies and models an Integrated System (IS) devoted to the HAH management at the operational level. The IS is designed to monitor the daily living of the apartment inhabitant, detect possible troubles and accidents, communicate with family, doctors, and emergency services. A Petri net model in a modular approach is proposed to describe the actions and the activities of the IS effectively. Nabih et al. does observe only simple aspects of smart home and not a smart building.

The Health At Home (HAH) is an alternative to the traditional hospital to promote early discharge and help patients and older adults live autonomously. Fanti et al. [30] specifies and models an Integrated System (IS) devoted to the HAH management at the operational level. The IS is designed to monitor the daily living of the apartment inhabitant, detect possible troubles and accidents, communicate with family, doctors, and emergency services. Fanti et al. [32] deals with the energy consumption management problem in buildings by modeling and controlling the major electric appliances. Renewable energies are considered by considering the production schedules of both wind and solar sources. Each appliance is described by modular mathematical models using the Matlab/Simulink software. A simulator is designed that models the load energy consumption and helps to recognize how they contribute to peak demand. In the proposed control strategy, the comfort conditions are respected for each appliance based on user preferences. Fanti et al. focus on energy and not performance related to time and drop rate.

Garcia et al. [31] use Petri nets to model Activities of Daily Living (ADLs) to capture the complex behaviors of ambient systems, such as the activities described above. Petri nets models’ granularity allows a complete and detailed understanding of the different variations and cases of the ADLs modeled. Garcia models the austere environment of the smart home and not complex parameters such as sensor grouping by locations. Ajao et al. [6] designed a smart room with automated window control, which can automatically open and close based on changing weather conditions. The response time and error percentage metrics were observed. Ajao et al. [6] observed the response time, the main metric studied in this article.

Casado et al. [36] focus on the problem of fault-tolerant maintenance of a networked environment in the domain of the Internet of things. Based on continuous-time Markov chains, together with a cooperative control algorithm, novel feedback model-based predictive hybrid control algorithm is proposed to improve the maintenance and reliability of the Internet of things network. Virtual sensors are substituted for the sensors that the algorithm predicts will not function properly in future time intervals; this allows for maintaining reliable monitoring and control of the Internet of things network. Casado et al. focus on maintenance and reliability and not edge capacity planning.

Some previous works adopted Petri nets to represent the data flows in a system for availability evaluation, mainly. Some others used queuing Petri nets for performance evaluation but not at a detailed level. Very few works presented comprehensive performance evaluation with detailed sensitivity analysis using DoE to assimilate the impact of different factors on the system performance, especially using queuing network models. As above mentioned, refs. [37,38] are the most related works that presented the use of queuing models for comprehensive performance assessment of data transactions and services in IoT infrastructures. We propose to use a queuing network-based message exchange architecture to comprehend the exact performance behaviors and evaluation of the data transactions. We employ a common type of queuing model but extensively construct a queuing network to represent the data transactions in an edge/fog based IoT infrastructure for smart buildings.

Table 1 presents a comparison of the collected studies, highlighting the application context, the metrics used, resource capacity analysis, sensor grouping by location, and number representation of cores per machine. Then, the works are discussed in a grouped way according to each comparison criterion.

Main components of the architecture-Some works use sensors to seek improvements in the elderly quality of life, monitoring them non-invasively to detect domestic accidents. Although the cited works have observed the communication of messages to external environments, the authors do not highlight how communication is carried out, nor do they focus on remote processing. Our proposal exploits the IoT and the edge and fog layers as complementary features that help optimize data processing. Metrics-Performance metrics facilitate understanding how the system behaves in different usage scenarios. These adopted metrics in our work are important for analyzing whether the system is functioning properly. The mean response time is important to verify that the configuration results in satisfactory transmission and processing time. The drop rate allows observing the number of requests that are discarded according to the network configuration. The flow rate shows the rate of traffic through the system. Using computational resources allows observing the configuration necessary to meet the system’s needs, avoiding the overload or resource idleness. The number of jobs in the system shows the number of requests in the system’s queues. Furthermore, this work is not limited to evaluating sensors or specific environmental conditions. The queuing network based message exchange architecture is configurable for any type of data collected by a set of sensors. Resource capacity analysis refers to evaluating how the system behaves according to the number of available resources. This analysis allows predicting whether the system will satisfy requests satisfactorily and avoiding wasting computational resources. We analyze the system’s behavior by changing the number of nodes in the fog in our work. Sensors grouped by location refers to how the model represents different sets of sensors. Our model allows us to assign different arrival rates depending on location. In our model, these locations can be seen as rooms in a building. This feature aims to make the model more realistic because, depending on the location, the data generated can be different. Our model is also the only one that represents the number of processing cores per machine. The fog layer has multi-core machines. The more cores a node has, the more requests it can process in parallel. The model allows for varying the fog capacity by changing the number of machines and the number of cores in each one of them. We believe this feature is critical to accurately representing architectures.

## 4. Methodology

**Figure 2:**
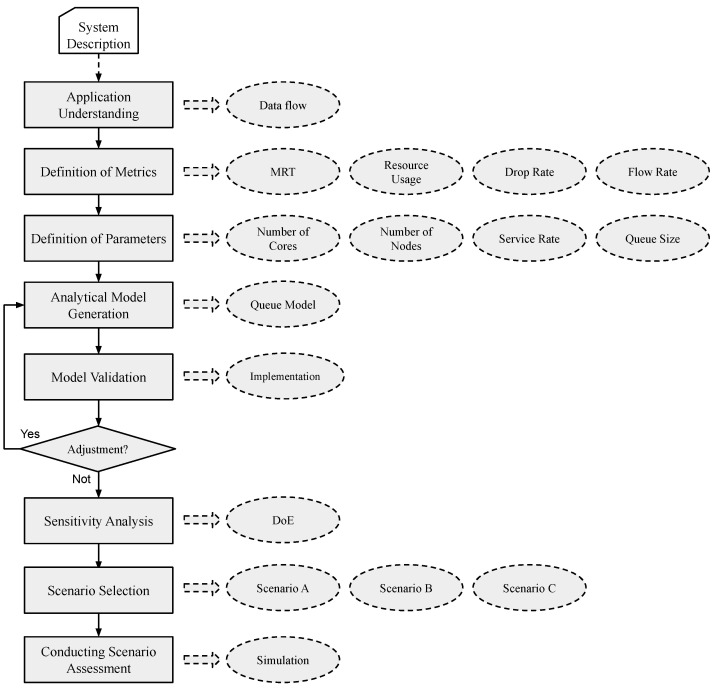
Analytical model development methodology.

The main objective of this work is to develop a queuing model that can evaluate IoT systems for intelligent buildings supported by edge and fog layers. To evaluate the model, some scenarios were built and based on them, sensitivity analysis and three simulations were developed to determine which factors most impact the metrics studied. Figure 2 presents a flowchart that summarizes the strategy used in this work.

**Application Understanding:** It is important to understand how the application works, define how many components are involved, and the system’s data flow, for example, where the data will be sent after passing through component ’x’. **Metrics Definition:** The metrics of interest must be identified, considering the model’s information to diagnose system performance. In this work, the selected metrics can be important in the end user’s perception and useful for system administrators, they are: MRT, resource utilization, discard rate, and throughput. **Definition of Parameters:** The parameters that will be inserted in the model are defined. These parameters define the behavior and capability of features of each component. In this work, the parameters added were the number of cores, nodes, service rate, and queue size. **Analytical Model Generation:** A performance model using queuing model is developed. In this part, the model is built considering the defined metrics and parameters and the expected results. The choice of the queuing model is because the considered intelligent building scenario has few components and the queuing model satisfactorily meets low complexity systems. **Template Validation:** Implementation of model validation using programming language considering the components inserted in the model. The results collected in the validation are compared with the results returned by the model, if they are similar values, the model is validated. Otherwise, there will be a need to adjust the model. If, after validation, the need to adjust the model is detected, it must return to the analytical model generation step. **Sensitivity Analysis:** Using DoE, the analysis presents results considering predefined factors and levels. From this, it is possible to identify the factors most relevant to the results of the chosen metrics and how the interaction between the factors and variations in their levels impact performance. **Scenario Selection:** Some scenarios are built for performance analytics. In this part, will be defined which scenarios can represent the reality of an intelligent building. The scenarios will be chosen to analyze the most important factors considering the sensitivity analysis results. **Conducting the Scenarios Assessment:** The built scenarios are evaluated using the queuing model through simulation. In each scenario, the factors will vary, and the chosen metrics will be analyzed, allowing to see which configurations the system has satisfactory performance.

## 5. System Architecture

This section presents the architecture of infrastructure for security monitoring in smart buildings. The architecture is discussed considering three aspects: general system architecture, message life cycle, and assumptions.

**Figure 3:**
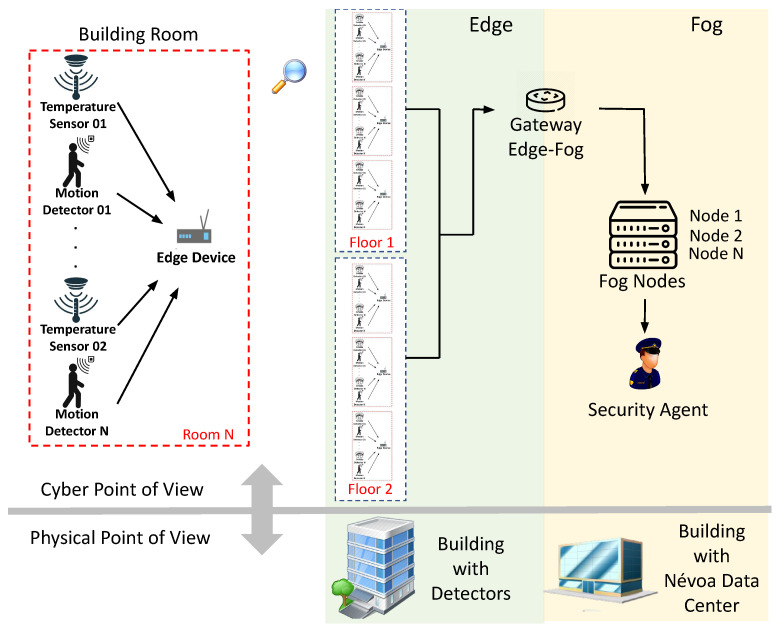
Overview of an architecture that allows you to monitor multiple sensors in an intelligent building.

General system architecture-Figure 3 illustrates the architecture of the system. Sensors can be different and have different purposes, but we exemplify some possible types of sensors as an illustration. The building can detect unexpected movement in environments (for example, when access to certain places is not allowed) and notify security guards. In addition, it can detect signs of fire and notify security agents to call the fire department. Security cameras can perform facial recognition through systems equipped with artificial intelligence. Such images can be compared with the database and check criminal records of passersby in the building. The architecture is composed of two computing layers: (i) edge computing layer in building rooms, for the integration of security sensor data; (ii) fog computing layer in data centers for internal client access (e.g., security agents). The edge computing layer enables real-time monitoring and aggregation of security data, using temperature and motion sensors to periodically collect and process data about each room’s environment. The edge computing layer is designed with multiple nodes to collect and process data in every room on every building floor. The fog computing layer is composed of: an edge-fog gateway for data grouping and load balancing between the two tiers; a set of fog nodes for parallel data processing; and a station where data is made available for internal client access.

Message life cycle-The architecture also indicates the life cycle of the data packages and the operational behavior of the security monitoring system. Data is periodically collected by motion and heat detectors and then sent to an edge device to be grouped and encapsulated as a security alert. If the device is busy, the data can be added to the queue, where they will be answered according to the order of arrival. However, if the edge device queue is completely occupied, the data will be discarded. These alerts are then transmitted to the fog via a gateway. The Edge-Fog gateway plays a role in data distribution and load balancing to the fog nodes. Load balancing is performed so that all fog nodes receive the same amount of processing requests, which is important to avoid overloading and queuing one node while other nodes are available. Messages are processed in fog nodes by specialized applications, which are customized for the alert type. As with the edge device, fog nodes also have a queued request limit, and if this limit is reached, the data will be discarded. Data processed in the fog layer is delivered directly to internal customers (security officers). Given this, questions may arise such as: “What are the impacts of requests’ arrival rate on the performance metrics of a smart building system?”; and “How does a specific resource capacity configuration impact the performance metrics of a smart building system?”.

Assumptions and Arguments-Some assumptions about the architecture under consideration are provided below to simplify the modeling.

- Edge layer
  − b1: Data generation has been modeled for all active sensors in a room, connected to an edge device that is also installed in the room.
  − b2: We did not take into account the communication latency between sensors and edge devices. In practice, the connection is formed by wireless communication. However, we have simplified the negative impact of short-haul communication at the edge layer on overall performance metrics.
  − b3: The communication latency of the connection between the edge and fog layers is assumed as a delay in the propagation of data from each edge node to the fog layer.
  − b4: The data collection of each sensor is independent of that of others. However, the rate of each input is deterministic, that is, with a fixed rate.
- Fog Layer
  − n1: We do not consider sophisticated load balancing in the fog layer. Jobs received at the fog gateway are evenly distributed to each of the nodes in the fog layer. To simplify the modeling, we did focus on the load balancing problem.
  − n2: We consider nodes with equal configurations, but the model allows the appraiser to configure the nodes in a heterogeneous way.
- IoT Infrastructure
  − i1: The performance of data transactions between IoT sensors and internal clients (security agents) is the main focus of the modeling, as in the work of Ashraf et al. [3]. Therefore, the involvement of physical components and their operational availability is minimized. We did not consider component failure and recovery behaviors in performance evaluation modeling.
  − i2: Our main focuses were (i) exploring the bottleneck in real-time security data transmission and (ii) exploring the impact of changing the fog layer configuration on performance metrics.

## 6. Queuing Network Model

**Figure 4:**
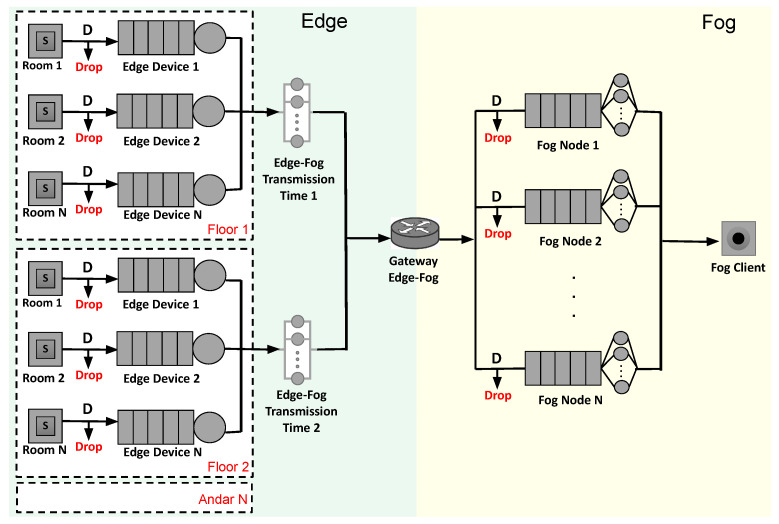
A queuing network based message exchange architecture of a smart building multi-layer architecture.

Queuing theory is a powerful analytical model that can represent complex systems [24,27,42,43]. This section presents a model based on queuing theory for the presented architecture, which is illustrated in Figure 4. The model has multiple entry points and one exit point. The Java Modeling Tools (JMT) was used to model and evaluate the proposed scenario. JMT is an open-source toolkit for analyzing and evaluating the performance of communication systems based on the queuing theory [44]. JMT was run in its 1.1.1 version through a *.jar* file in a Linux Ubuntu 18.04 LTS environment. The model (with a .jsimg extension) can be downloaded through the following url: https://tinyurl.com/queuemodel, (accessed on 2 July 2021). Table 2 describes all the model elements.

Data flow in the model occurs from left to right. The multiple entries in the model correspond to the rooms in the building. Each room has sensors that generate requests within a predefined time interval and an edge device that acts as a gateway between the edge and the fog layer. Such an edge device is represented by a queue and a unique internal server. The rooms have *n* sensors, an amount that can vary depending on the size of the room, as very large rooms may need several sensors to cover their entire area. The arrival rate will depend on the number of sensors and the data generating distribution. When we increase the arrival rate, more sensors are in operation as they are calibrated for a fixed generation interval. When the arrival rate is higher than the system can handle, the data is discarded. It is considered that the rooms are organized into floors. Each floor has a certain distance from the fog layer. This way, there is a delay (“Edge-Fog Propagation Time”) from each floor to the fog layer. Propagation time components do not have a specific service; it is just a component that causes a delay in propagating a request, emulating a network delay.

In queuing theory, Kendall notation is popularly used as a standard system for the description and classification of a queuing node. Originally, queuing models were represented using three factors as A/S/c (A indicates the arrival time of between each item to the queue, S represents the distribution of service time, and c is the number of vacant slots in the queue). An extension of the above representation is A/S/c/K/N/D in which K represents capacity, N indicates job population size, and D reflects the discipline of the queue node. In this work, we adopt this representation in building the queuing network for the edge/fog system. In the fog layer, there is a gateway that is used as an entry point. Upon arriving at the gateway, messages can be distributed following a specific load balancing strategy. In this work, in the simulations section, we consider the equal distribution strategy. By Kendall notation, the network follows the D/M/c/K/FCFS pattern. The generation rate follows a deterministic pattern (*D*), as the sensors are calibrated for a fixed generation interval. However, node service times follow an exponential distribution. Service stations have a number (*c*) of servers, which we consider here as CPU cores. It is important to note that the system has a limited queuing size, exceeding requests being dropped. The respective queues have a fixed size k, and all queues together sum K total size. The arrival policy is first to come, first service (FCFS). The fog also has a *sink* station (“Fog Client”) corresponding to the location where security agents can access sensitive data in real-time.

## 7. Sensitivity Analysis with DoE

Sensitivity analysis is a measure of the effect of a given input data about the output data, aiming to outline the weak links of the computer systems, and from then on, seek to adopt a set of techniques that aim to improve these systems in different scenarios [45]. Some jobs use sensitivity analysis to provide the necessary security and forward the perspective of system administrators [46,47]. In this work, we have applied a sensitivity analysis with DoE.

The Design of Experiments (DoE) corresponds to a collection of statistical techniques that deepen the knowledge about the product or process under study [48]. It can also be defined by a series of tests in which the researcher changes the set of variables or input factors to observe and identify the reasons for changes in the output response.

System designers often adopt sensitivity analysis to evaluate how “sensitive” a metric is to changes in the model [49]. The parameters to be changed are defined using an experiment plan. The goal is to generate the most significant amount of information with the least possible experiments. The behavior of the system based on parameter changes can be observed using sets of outputs. In the literature, there are three categories of graphs usually adopted for experiments with DoE:

- Pareto chart is represented by bars in descending order. The higher the bar, the greater the impact. Each bar will represent the influence of each factor on the dependent variable.
- Main effects graphs are used to examine the differences between the level means for one or more factors, graphing the mean response for each factor level connected by a line. It can be applied when using a comparison between the relative strength of the effects of various factors. The signal and magnitude of the main effect can express the mean response value. The magnitude will express the strength of the effect. The higher the slope of the line, the greater the magnitude of the main effect. It is necessary to consider that the horizontal line has no main effect; each level will affect the response in the same way.
- Interaction graphs are responsible for identifying interactions between factors. An interaction occurs when the influence of a given factor on the result is altered (amplified or reduced) by the difference in another factor’s level. Assuming the lines on the graph are parallel, there is no interaction between the factors. If they are not parallel, there is an interaction between the factors.

This section describes the experiments performed to analyze factors that can influence the performance of a smart building. It was also analyzed how changes in their levels impact the performance of the system. The results obtained are discussed based on the mean response time metric.

### 7.1. Design

The mean response time (MRT) metric is analyzed through the DoE. The choice of MRT is due to its more direct impact on the perception of the end-user. Resource utilization level, for example, is a metric considered to be of secondary type. Four factors were adopted in this study: service rate, number of nodes, number of cores, and queue size. All factors have two levels. The service rate factor refers to the rate it takes the server to execute a request, and its levels are 0.033 msg/ms and 0.044 msg/ms. The number of nodes refers to the number of servers in the fog, defined as 5 and 10. The number of cores is defined by the number of cores in each fog server, defined as 2 and 4. Queue size refers to the number of requests that will be added to the server queue. Its two levels are 50 and 100. Table 3 summarizes the factors and levels chosen to perform the DoE using the MRT metric. They must be combined to define how the experiments should be performed with all factors and levels defined.

### 7.2. Results of DoE

**Figure 5:**
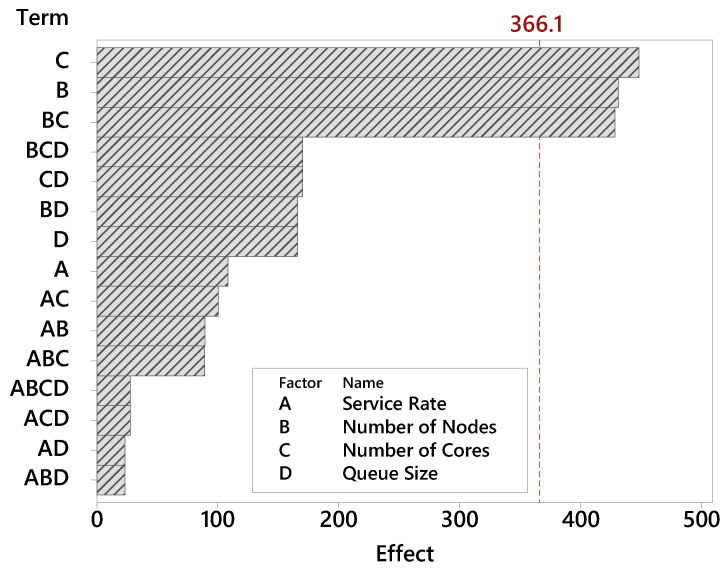
Influence of factors on the MRT metric.

The Pareto chart determines the magnitude and importance of factors. Figure 5 presents the Pareto graph for the factors related to the MRT metric. When a factor has a high impact on the tests, very different values are obtained when changing its level. Bars that cross the red reference line (effect 366.1) are considered statistically significant. These factors are statistically significant considering the 95% statistical confidence with the terms of the current model. The factor number of cores has the greatest relevance among the factors in this study. Therefore, the number of cores by fog nodes is decisive in the building’s monitoring efficiency. The number of nodes factor also has high relevance. Queue size and service rate proved to be far less influential. As the Pareto plot displays the absolute value of the effects, you can determine which effects are large, but it cannot determine which effects increase or decrease the response time.

**Figure 6:**
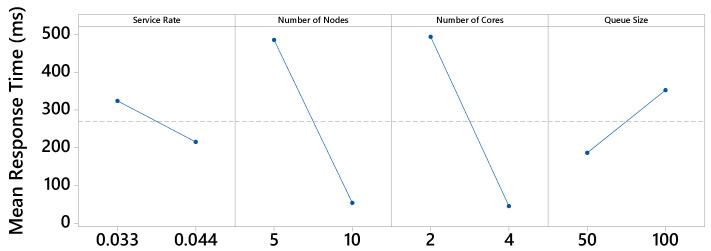
Main effects for MRT.

Figure 6 presents the main effects graph for the MRT metric. The graph breaks down the average resulting values for each level. The more horizontal the line, the less influence that factor has, as it means that the different levels of the factor similarly influence the result. All factor levels interfere with the MRT metric in some way. The factors number of nodes and number of cores have the greatest effect. Regarding the number of nodes factor, with 5 nodes, the highest mean response time was obtained (485 ms), while with 10 nodes, this time was much lower (53 ms). Therefore, the building will be more efficient using 10 nodes in the fog. Regarding the number of cores factor, it can be seen that the MRT is much higher when using 2 cores instead of 4. When using 4 cores, the processing speed is doubled. Thus, the MRT is drastically reduced.

**Figure 7:**
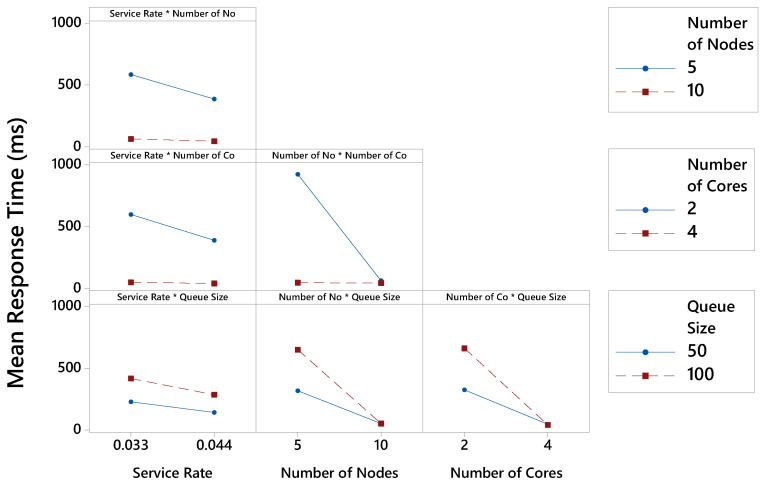
Interactions of factors considering the MRT. The * mark means the relationship between two factors.

Figure 7 displays the interactions for each possible factors combination. Two factors interact with each other if the effect depends on the variation in the effects of the other. There is an interaction between all factors, although the variation of effects is low in some cases. In the interaction between service rate and the number of nodes, the largest rate variation occurs when nodes are equal to 5. When the service rate equals 0.033 msg/ms, the MRT reaches 584 ms. With a service rate of 0.044 msg/ms, the MRT is equal to 386 ms. In the interaction between service rate and the number of cores, the greatest rate variation occurs when cores are equal to 2. The MRT is equal to 598 ms when the service rate reaches 0.033 msg/ms and 389 ms when the rate is 0.044 msg/ms. The interaction between service rate and queue size is relatively low (compared to other interactions), with no variation in the MRT when changing the levels of these factors. In the interaction between the number of nodes and cores, the greatest variation occurs when the number of cores equals 2. With 5 nodes in the fog, the MRT reaches 923 ms. With 10 nodes, the MRT is equals 63 ms. In the interaction between the number of nodes and queue size, the greatest variation occurs with a queue size equal to 100. The MRT reaches 651 ms for 5 nodes, and with 10 nodes, the MRT equals 53 ms. In the interaction between the number of cores and queue size, the greatest variation also occurs when the queue size equals 100. With this configuration, MRT reaches 661 ms when nodes have 2 cores. With 4 cores, the MRT is equal to 43 ms.

## 8. Simulations Results

This section presents the simulations performed on the proposed queuing network based message exchange architecture considering the variation in the two factors that most influenced the performance of the building monitoring system. These factors were defined based on the DoE performed previously, and they are the number of nodes in the fog and the number of cores in each node. Table 4 presents the configuration used for the experiments. The tag **X** indicates that the component has no queue capacity definition. The time column represents the service time for the queue components. The propagation time of components represents the time for communication between one layer and another. In this section, three scenarios are presented: Scenario A presents a variation under the number of nodes; Scenario B presents a variation under the number of cores; and Scenario C examines both factors together.

The simulation follows a discrete-event simulation (DES) model with the operation of a system as a (discrete) sequence of events in time. Each event occurs at a particular instant in time and marks a change of state in the system. Between consecutive events, no change in the system is assumed to occur; thus, the simulation time can directly jump to the occurrence time of the next event, which is called the next-event time progression.

### 8.1. Scenario A: Varying the Number of Cores

In this scenario, the factor number of cores was varied, which obtained the greatest relevance according to the DoE. The number of cores in the fog was varied in 1, 2, 4, and 8 cores, while the number of cores in the edge nodes remained fixed (1 core). The amount of edge and fog nodes was fixed at 6 and 5 nodes, respectively.

**Figure 8:**
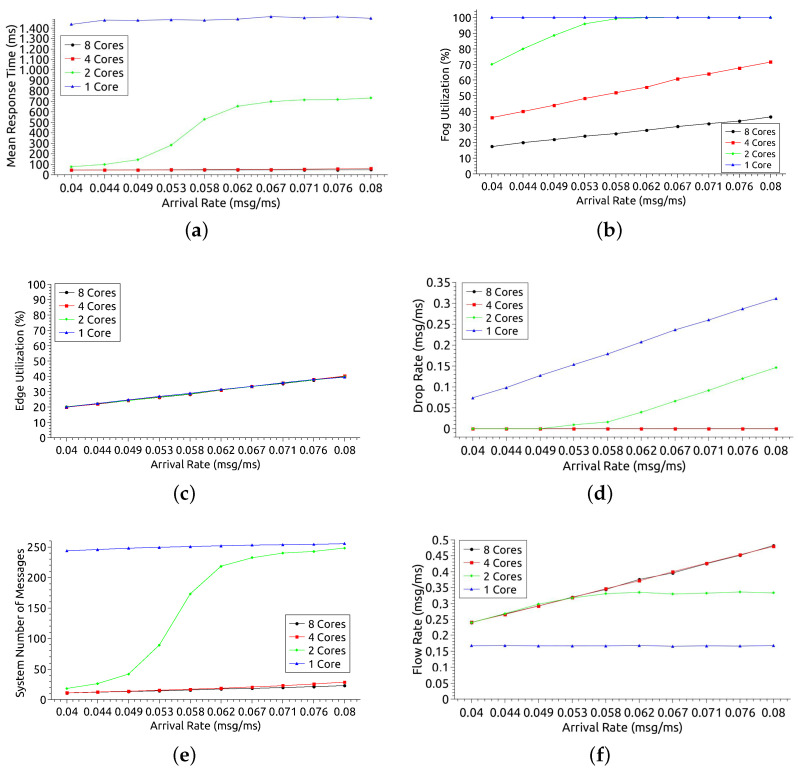
Model analysis results considering different numbers of cores. (**a**) Mean Response Time (MRT); (**b**) Fog Utilization; (**c**) Edge Utilization; (**d**) Drop Rate; (**e**) System Number of Messages; (**f**) Flow Rate.

Figure 8 presents the results considering different numbers of cores in the fog nodes: 1, 2, 4, and 8. Figure 8a shows the MRT of the entire system. The greater the resources, the smaller the MRT tends to be, but this pattern is not always apparent. Thus, when there is only 1 core, the MRT is much higher than the other configurations, even for low arrival rate values. With 1 core, it is noted that the growth remains stagnant between 1400 ms and 1500 ms. Such stagnation occurs due to the high utilization of resources with little processing power (see Figure 8b). The stagnation also happens with 2 cores, but only from AR = 0.062 msg/ms, reaching an MRT of approximately 700 ms. With 4 and 8 cores, the MRTs obtained are very close. This proximity suggests that it is perhaps more advantageous and cost-effective to use just 4 cores. Considering the smallest AR (0.04 msg/ms), we have an MRT of 1434 ms, 77 ms, 46 ms, and 45 ms for 1, 2, 4, and 8 cores, in that order. Assuming the desired MRT of around 100 ms, the configurations that meet this restriction are those with 2 or more cores. Considering the extreme point (AR = 0.08 msg/ms), there is an MRT of 1492 ms, 731 ms, 59 ms, and 47 ms for 1, 2, 4, and 8 cores, respectively. Assuming the 100 ms SLA, such time constraint would be met with 4 or 8 cores.

Figure 8b shows the fog utilization. The greater the number of cores, the lower the use of resources tends to be. Thus, the use with 1 core is much higher than with 8 cores, even with low AR values. When fog nodes have only 1 core, utilization is 100% for all analyzed arrival rates. When there are 2 cores, the utilization for AR = 0.04 msg/ms is approximately 70% and reaches 100% AR ≥ 0.058 msg/ms. With 4 and 8 cores, utilization grows as the arrival rate increases, but it does not deplete available resources. Considering the point of least demand (AR = 0.04 msg/ms), we have a utilization rate of 100 %, 70 %, 36 % and 17 % for 1, 2, 3 and 4 cores, in this order. Assuming an SLA premise where maximum utilization of 80% is accepted, such restriction can be met when the main fog nodes are configured with 2 or more cores. Considering the extreme point (AR = 0.08 mg/ms), is observed a utilization of 100%, 100%, 71% and 36% for 1, 2, 4 and 8 cores, respectively. With an SLA assumption of 80%, this option can be attended with 4 or 8 cores in each fog node.

Figure 8c shows the edge utilization. As the arrival rate increases, the edge utilization rate also increases. However, changing the number of cores in edge nodes does not influence the result because the edge does not depend on processing in fog nodes to perform its tasks. Regardless of the SLA premise on the edge utilization, choosing any number of nodes in the fog is possible. The number of fog nodes does not interfere with edge utilization.

Figure 8d shows the drop rate. The drop rate tends to decrease as the number of cores increases. Thus, configurations with 4 or more cores are well below the others when high arrival rates. With 1 core, the system has a drop rate of at least 0.1 msg/ms for low AR, and the rate increases as AR grows. With 2 cores, there is no message dropping until AR = 0.049 msg/ms, but with higher arrival rates, it can discard approximately 0.15 msg/ms. The system does not discard any messages for configurations with 4 and 8 cores, even with AR = 0.08 msg/ms. Considering the smallest AR (0.04 msg/ms), discard exists only when the system is configured with 1 core, with a drop rate equal to 0.07 msg/ms. Assuming the SLA requires no message dropout, the configuration needs to have 2 or more cores on each node in the fog. Considering AR = 0.08 msg/ms, there is a discard of 0.34 msg/ms and 0.14 msg/ms for 2 and 4 nodes, and no discard with 4 and 8 cores. For the SLA where there should be no discard, the configurations that meet this requirement have 4 or 8 cores.

**Figure 9:**
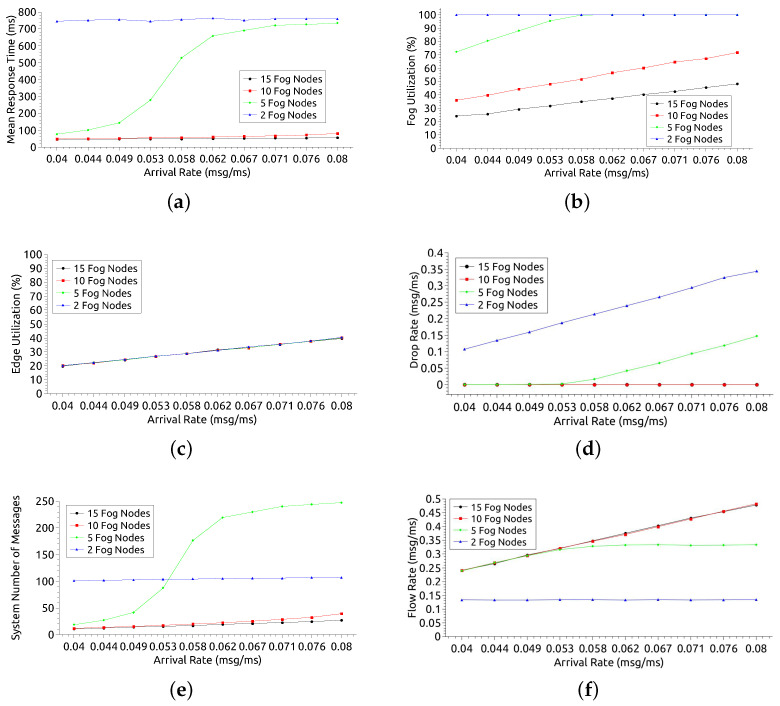
Model analysis results considering different numbers of fog nodes. (**a**) Mean Response Time (MRT); (**b**) Fog Utilization; (**c**) Edge Utilization; (**d**) Drop Rate; (**e**) System Number of Messages; (**f**) Flow Rate.

Figure 8e displays the number of messages across the system. Note that the lines of the graph have curves similar to those of the MRT (see Figure 9a). The greater the number of messages in the system, the longer the queue and the longer it will be serviced. In the 1 core configuration, the system always has approximately 250 messages, regardless of the arrival rate. When there are 2 cores, the number of messages is less than 50 until AR = 0.049 msg/ms. For 4 and 8 cores configurations, messages are less than 50 for all arrival rates.

Figure 8f shows the system flow rate. Throughput is expected to increase as the arrival rate increases because more messages arrive. It can be observed that such behavior in configurations with 2, 4, and 8 cores. However, an inflection point with 2 cores and AR = 0.58 msg/ms is observed when the flow stabilizes. The 1-core configuration has a much lower flow rate than the others and remained stable for all arrival rates. Stability happens because the system becomes overloaded and starts dropping messages (see Figure 9d). Considering AR = 0.04 msg/ms, the flow rate is 0.16, 0.23, 0.24 and 0.24 for 1, 2, 4 and 8 cores, in that order. Assuming an SLA premise where a flow rate greater than 0.2 msg/ms is needed, it is possible to meet this restriction with 2 or more cores. Considering AR = 0.08 msg/ms, the flow rate is 0.16, 0.33, 0.47 and 0.48 for 1, 2, 4 and 8 cores, respectively. Considering a minimum flow rate SLA of 0.04 msg/ms, the configurations that meet this requirement have 4 or 8 cores in each node in the fog.

In conclusion, the edge is not impacted by the change in the number of cores in the fog. The edge does not depend on fog processing to function. For MRT metrics, the system behaves stably in all fog configurations except with 2 cores. With this setting, from AR = 0.053 msg/ms, the MRT increases because fog resources also grow, getting close to 100%. The increase in MRT also increases the number of messages in the system, making the graphs of the two metrics similar. Also, the drop rate is only existing when fog nodes have only 1 or 2 cores.

### 8.2. Scenario B: Variation in the Number of Nodes in the Fog

The second most relevant factor in the DoE was varied in this scenario: the number of nodes in the fog. The number of nodes in the fog was varied in 2, 5, 10, and 15 nodes, while the number of nodes on edge remained fixed (6 nodes). Each edge node has 1 processing core, while fog nodes have 2 cores.

Figure 9 presents the results considering a different number of nodes in the fog. Figure 9a shows the MRT of the entire system for the 4 variations: 2, 5, 10, and 15 fog nodes. It is expected that the larger the number of nodes, the smaller the MRT, as the system performs load balancing. This behavior can be seen in the configurations observed in this analysis. However, with 10 or more nodes in the fog, the difference in MRT may not be significant. When the resources were reduced to 5 and 2 nodes, the MRT was much higher due to AR, reaching 734 ms and 749 ms, respectively. The MRT’s proximity for 10 and 15 nodes suggests that it might be more advantageous and cost-effective to use just 10 nodes in the fog. Regarding the arrival rate growth, it is observed that the AR has a greater impact when there are 5 fog nodes. The MRT has little significant growth for 2, 10, and 15 nodes as the workload increases. However, with 2 nodes, the MRT is always above 700 ms, regardless of the arrival rate. A pattern observed in the four scenarios is about the stagnation of MRT growth as a function of AR. In all configurations, there is a certain stability in the MRT. However, for 5 nodes, this could only be observed with AR from 0.071 msg/ms. The range AR = [0.071 msg/ms–0.08 msg/ms] can be used to offer infrastructure customers an average MRT of 700 ms. For 15 nodes, this average would be 55 ms. Considering the smallest AR (AR = 0.04 msg/ms), there is an MRT of 744 ms, 77 ms, 49 ms, and 46 ms for 2, 5, 10, and 15 nodes, respectively. Considering a Service Level Agreement (SLA) assumption that reports a requirement of MRT > 100 ms, such a time constraint would be met with configurations from 5 nodes in the fog. Considering the extreme point, with the highest demand (AR = 0.08 msg/ms), there is an MRT of 759 ms, 734 ms, 81 ms, and 55 ms for 2, 5, 10, and 15 nodes, respectively. For a 100 ms SLA assumption, this time restriction will only be met from 10 fog nodes.

Figure 9b shows the fog utilization rate. The greater the number of nodes in the fog lower tends to be the use of resources. Thus, the utilization with 2 cores is much higher than with 5, 10, and 15 nodes. A pattern observed in all scenarios except for 2 nodes is that utilization grows as the arrival rate increases. With 2 cores, usage growth remains stable at 100% for all arrival rates. When there are 5 nodes, utilization grows until reaching 100%, with AR ≥ 0.058 msg/ms. With 10 and 15 nodes, the highest utilization achieved is 71% and 48%, respectively. Considering the smallest AR (0.04 msg/ms) there is a utilization of 100%, 72%, 36% and 24% for 2, 5, 10 and 15 nodes, in that order. An SLA that considers utilization of up to 80% can be met with configurations with 5 or more nodes. Considering the point of highest demand (AR = 0.08 msg/ms), the utilization rate is 100%, 100%, 71% and 48%, respectively. For the usage SLA premise up to 80%, this restriction can be met from 10 nodes in the fog.

Figure 9c shows the edge utilization rate. As expected, as the arrival rate increases, the edge utilization rate also increases. However, changing the number of nodes in the fog does not influence the result much. The edge does not rely on processing on fog nodes to perform its tasks, as this layer comes before the fog layer. Regardless of the preference for using the edge, the system designer can choose any number of nodes in the fog, as this does not interfere with using the edge resources.

Figure 9d shows the drop rate of messages. The greater the system capacity, the lower the drop rate tends to be. Thus, the drop rates for configurations with 10 nodes or more are well below the others when the arrival rate is high. The system does not discard any messages for configurations with 10 or more nodes, even with AR = 0.08 msg/ms. With 5 nodes, there is no message discard until AR = 0.053 msg/ms, but with higher arrival rates, it can discard more than 0.1 msg/ms. With 2 nodes, the system has a drop rate of at least 0.1 msg/ms for low AR, and the rate increases with AR growth. Considering the smallest AR (0.04 msg/ms), discard only when the system is configured with 2 fog nodes, with a drop rate equal to 0.1 msg/ms. Assuming an SLA requires no discard, the configuration must have 5 or more nodes in the fog. Considering the point of greatest demand (AR = 0.08 msg/ms), there is a discard of 0.34 msg/ms and 0.14 msg/ms for 2 and 4 nodes, in that order, with no discard for 10 and 15 nodes. Imagining the SLA where there should be no discard, the configurations that meet this requirement have 10 or more nodes.

Figure 9e displays the number of messages (requests) in processing state within the system.The lines of the graph have curves similar to those of the MRT graph (see Figure 9a). This similarity occurs because the number of messages in the system is related to the MRT. When the MRT increases, there will have more messages in the system because the queues get longer. However, with 2 nodes in fog, the observed MRT is higher than with the 5-node configuration. Still, for the configuration with 2 nodes, there are fewer messages in the system as of AR = 0.58 msg/ms. The low number of messages with 2 nodes is due to the higher drop rate than the other configurations, as it has less processing capacity and fewer queues (see Figure 9d).

Figure 9f shows the system flow rate. The flow rate is expected to be directly proportional to the arrival rate. The same behavior can be observed with configurations including 5, 10, and 15 nodes. However, there is an inflection point with 5 nodes and AR = 0.58 msg/ms when the rate stabilizes. The 2-node configuration has a much lower flow rate than the others and remained stable for all arrival rates. Stability happens because the system becomes overloaded and starts dropping messages (see Figure 9d). Considering AR = 0.04 msg/ms, the flow rate is 0.13 msg/ms, 0.23 msg/ms, 0.24 msg/ms and 0.24 msg/ms for 2, 5, 10 and 15 cores, in that order. Assuming an SLA premise where a flow rate greater than 0.2 msg/ms is needed, it is possible to meet this restriction with 5 or more nodes. When AR = 0.08 msg/ms, there is a flow rate of 0.13 msg/ms, 0.34 msg/ms, 0.47 msg/ms and 0.48 msg/ms for 2, 5, 10 and 15 nodes, respectively. Considering an SLA of at least 0.04 msg/ms for flow rate, the configurations that meet this requirement are those that have 10 or more nodes in the fog.

Analyzing the results of this section more broadly, it can be seen that the use of the network edge is not impacted by the change in the number of nodes in the fog. For the MRT metric, the system behaves stably in all fog configurations except with 5 nodes. With that setting, as of AR = 0.053 msg/ms, the MRT has considerable increases. In this case, the use of fog resources also grows, getting close to 100%. The increase in MRT causes the number of messages in the system also to increase. With AR from 0.058 msg/ms, the configuration with 5 nodes starts to have more messages in the system than the configuration with 2 nodes. With 2 nodes, the system has the lowest flow rate and the lowest processing capacity, which also means shorter queues, causing an increase in the drop rate.

### 8.3. Scenario C: Varying the Number of Nodes and Cores Simultaneously

In the previous scenarios (A and B), the factors number of fog nodes and number of cores in each node were analyzed separately. Such analyzes allowed us to observe how each factor interferes in all metrics in a very detailed way. However, in addition to having an isolated impact on the system behavior, the DoE analysis showed that there is a strong interaction between both factors on the mean response time, as shown in the Pareto graph (Figure 5) and interaction graph (Figure 7). Such graphs only indicate the existence and magnitude of the interaction, but not accurately. Therefore, this section shows the variation of the two factors with values equal to 1, 2, 4, and 8 nodes/cores. The number of nodes at the edge was fixed with 6 nodes with 1 core each. Table 5 presents the combinations between the factors analyzed in this scenario.

**Figure 10:**
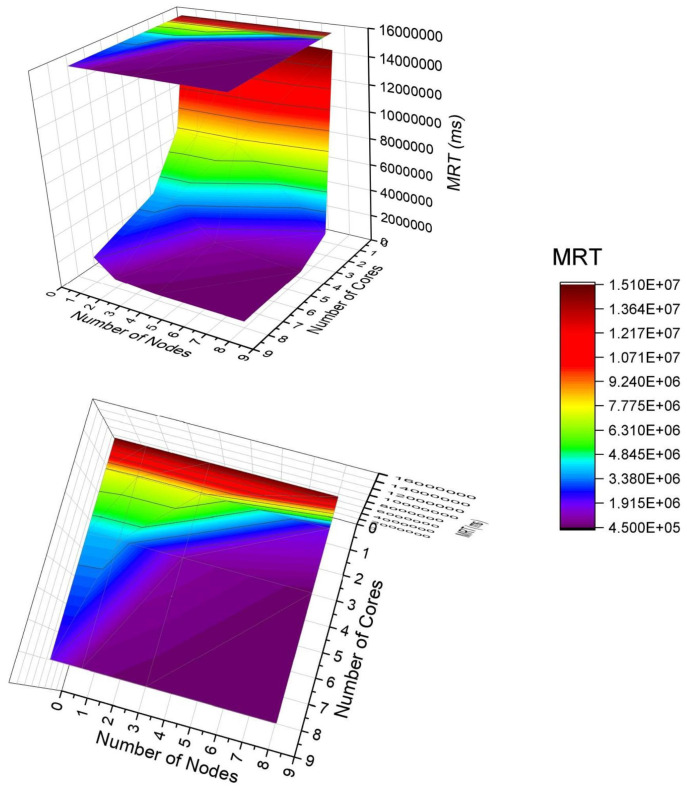
Analysis of mean response time by varying factors simultaneously.

Figure 10 presents a 3D surface graph to show the behavior of the system considering the MRT by varying two factors with a high impact on performance. First of all, it is essential to say that the colors are related to the MRT result. The bar on the right indicates the magnitude of the results. The top indicates larger MRTs, and the bottom indicates smaller MRTs. Therefore, the purple color represents the lowest MRT, and the red color means the highest MRT. In the graph, it is worth noting the presence of a projection at the top that facilitates the observation of the interaction of factors. Changing the number of cores is greater than the impact of changing the number of nodes. The purple is present for most of the projection. The purple corresponds to MRTs in the top place of 1.9 $\times \;10^{6}$ ms—that is, if adopting any node number with core number greater than 2, the MRT will be below 1.9 $\times \;10^{6}$ ms. Observing the red part of the graph (larger MRT), the dominant factor is the number of cores. If this number is small, the MRT always tends to be high, and there is no point in changing the number of nodes. Therefore, the result indicates that it is often more beneficial to invest in the capacity of a single server node with greater processing power than to acquire several smaller servers. This case study shows that when purchasing an 8-node number with 8 cores, there will be the same performance as 6 nodes with 6 cores for this case study.

## 9. Conclusions

This work proposed a queuing (D/M/c/K/FCFS) network based message exchange architecture to evaluate the performance of smart building infrastructures. The architecture evaluated includes IoT sensors and edge-fog components. The model allows the analysis of several metrics, such as utilization level, drop rate, mean response time, and flow rate. In addition, the model has features that are not found in related works, such as resource capacity analysis, sensors grouped by location, and the number of cores per machine. Performance analyzes were performed considering the proposed queuing network based message exchange architecture through a sensitivity analysis using DoE and simulations. In the analysis with DoE, some factors that can impact building performance were studied: number of nodes available in the fog, number of cores on each node, queue size, and service rate. The two most relevant factors were explored in the simulations: the number of nodes in the fog and cores. The simulations results show that the arrival rate and the number of resources available in the fog can be very influential on system performance, As the arrival rate increases, it may be necessary to use more fog resources for the system to function satisfactorily. In the results for drop rate in scenario A, for example, when we have 2 processing cores and AR = 0.04 msg/ms, there was no message discard, but when AR > 0.049 msg/ms, the number of discarded messages grows with the arrival rate increment. This work can be useful for system designers in this context to better define building monitoring system configurations. The limitation of this work lies in the lack of cloud support. Therefore, we intend to extend the model, including the cloud layer and its components, evaluating new scenarios as future work. Furthermore, we intend to develop and implement a real system to compare its performance with the results obtained with the model.

## Figures and Tables

**Table 1:** Related Works.

Reference	Application Context	Metrics	Resource Capacity Analysis	Sensors Grouped by Location	Represents Number of Cores per Machine
[28]	Smart home	System effectiveness	Not	Not	Not
[29]	Smart home	System effectiveness	Not	Not	Not
[33]	Smart building	Energy efficiency	Not	Not	Not
[34]	Smart home	User comfort perception	Not	Not	Not
[30]	Smart home	System effectiveness, vivacity, limitation and reversibility.	Not	Not	Not
[32]	Smart building	Energy efficiency	Not	Not	Not
[31]	Smart home	System effectiveness	Not	Not	Not
[6]	Smart home	Error percentage, Response time.	Not	Not	Not
[35]	Smart building	Trajectory pedestrian prediction	Not	Not	Not
[36]	Smart building	Temperature	Not	Not	Not
[41]	Smart building	Indoor environment quality sensing	Not	Not	Not
[37,38]	Smart building	IoT services	Not	Not	Not
This Work	Smart building	Resource utilization, MRT, Drop rate, Flow rate, Number of jobs in the system.	Yes	Yes	Yes

**Table 2:** Description of model components.

Type	Element	Description
	Sensors	Responsible for data generation.
	Edge Device	Responsible for data pre-processing.
Processing	Gateway Edge-Fog	Responsible for forwarding data from the edge to the fog.
	Fog Node	It saves data, processes and forwards information and/or alerts to security agents.
Communication	Edge-Fog Propagation Time	Edge data propagation delay.
Execution Status	Fog Client	Represents the end of data processing and availability for security agents.

**Table 3:** DoE Factors and Levels.

Factors	Level 1	Level 2
Service Rate	0.033	0.044
Number of Nodes	5	10
Number of Cores	2	4
Queue Size	50	100

**Table 4:** Parameters Inserted in the Model.

Component Type	Component	Time (ms)	Queue Size
Queue Machine	Edge Devices	5	50
	Fog Nodes	30	50
Propagation Time	Edge-Fog 1	6	X
	Edge-Fog 2	12	X

**Table 5:** Combinations of the factors number of cores and number of nodes.

Combination	Number of Cores	Number of Nodes
#1	1	1
#2	1	2
#3	1	4
#4	1	8
#5	2	1
#6	2	2
#7	2	4
#8	2	8
#9	4	1
#10	4	2
#11	4	4
#12	4	8
#13	8	1
#14	8	2
#15	8	4
#16	8	8

## Data Availability

Not applicable.
